# Supplementary material for: An isoform quantitative trait locus in SBNO2 links genetic susceptibility to Crohn’s disease with defective antimicrobial activity
Source: Nat Commun. 2024 May 28;15:4529. doi: 10.1038/s41467-024-47218-3 (PMC11133462; doi:10.1038/s41467-024-47218-3)
Supplement: Supplementary file 1 — Supplementary Information [file 41467_2024_47218_MOESM1_ESM.pdf]

Supplementary Figure 1

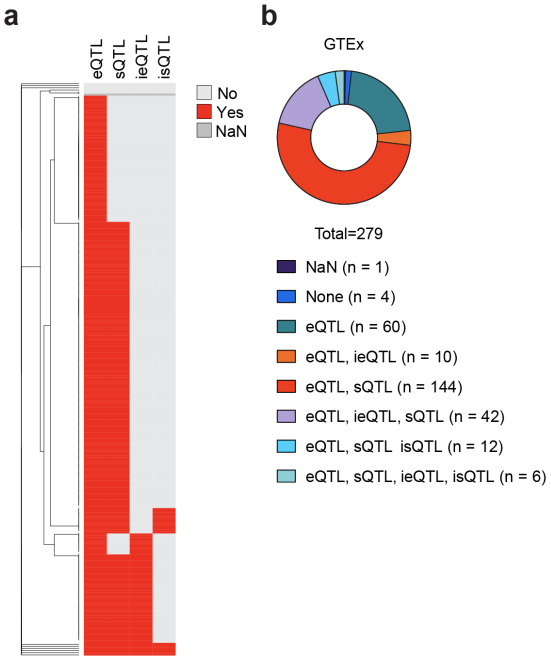

Supplementary Figure 1: GTEx eQTL, sQTL, ieQTL, and isQTL analysis of confident GWAS IBD genes. (a) Heatmap presentation of the presence or absence of GTEx eQTL, sQTL, ieQTL, and isoQTL across confident GWAS IBD genes[2] (n = 279, Bolton C. et al., Gastroenterology 2022, Supplementary Table 4: Genes included in the analysis of the intersection of monogenic IBD genes and polygenic IBD loci). (b) Pie chart presentation of numbers of confident GWAS IBD genes according to (a). Source data are provided as a Source Data file for Supplementary Figure 1a.

Supplementary Figure 2

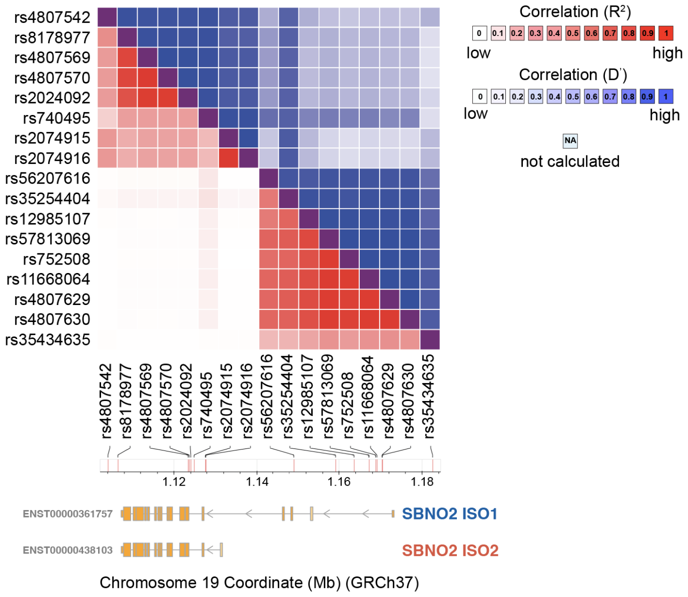

Supplementary Figure 2: LDlink linkage disequilibrium analysis of variants in *SBNO2* with GWAS-based association with IBD/CD.

Supplementary Figure 3

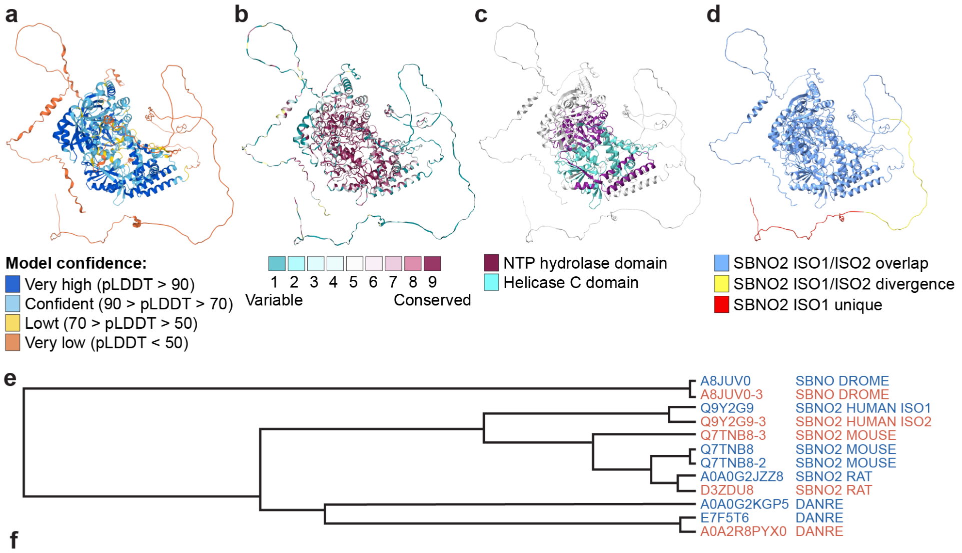

f

```
Q7TNB8      SBNO2 MOUSE            1 ---------------------------------------MLAVEPTMDGDFPPHEL-PPP  20
Q7TNB8-2    SBNO2 MOUSE            1 ---------------------------------------MLAVEPTMDGDFPPHEL-PPP  20
Q7TNB8-3    SBNO2 MOUSE            1 ------------------------------------------------------------   0
Q9Y2G9      SBNO2 HUMAN ISO1       1 ---------------------------------------MLAVGPAMDRDYPQHEP-PPA  20
Q9Y2G9-3    SBNO2 HUMAN ISO2       1 ------------------------------------------------------------   0
A0A0G2JZZ8  SBNO2 RAT              1 -------------------------------------MPTLATSVVMDTEDYLHPEGPQL  23
D3ZDU8      SBNO2 RAT              1 ---------------------------------------MLAVEPTMDGDFPPHEL-PPP  20
E7F5T6      DANRE                  1 ------------------------------------------------------------   0
A0A0G2KGP5  DANRE                  1 -------------------------------------MPSLSIPPAMDGHSFSNSVGPQL  23
A0A2R8PYX0  DANRE                  1 ------------------------------------------------------------   0
A8JUV0      SBNO DROME           181 KLNNMVTAMGSPAARSSGNAGTTGSSQGGAIGSTSSYLNSLTTNELMNLAAYVAA----- 235
A8JUV0-3    SBNO DROME           181 KLNNMVT----------------------------------------------------- 187

Q7TNB8      SBNO2 MOUSE           21 GG--GI----QLQNRLL----HCPWWGSFSPSL----YPTFSSENQQFVGSTPFLGGQSC  66
Q7TNB8-2    SBNO2 MOUSE           21 GG--GI----QLQNRLL----HCPWWGSFSPSL----YPTFSSENQQFVGSTPFLGGQSC  66
Q7TNB8-3    SBNO2 MOUSE            1 ------------------------------------------------------------   0
Q9Y2G9      SBNO2 HUMAN ISO1      21 GS--LLYSPPPLQSAML----HCPYWNTFSLPP----YPAFSSDSRPFMSSASFLGSQPC  70
Q9Y2G9-3    SBNO2 HUMAN ISO2       1 -----------------------------------------MREPLPGSASWGTPGPP  17
A0A0G2JZZ8  SBNO2 RAT             24 GSPKFSAPSPPLTSSMESQLYQPNSWAYSQQAQYNQHYPMQSGRQLQ-------------  70
D3ZDU8      SBNO2 RAT             21 GG--GI----QLQNRLL----HCPWWGSFSPPL----YPTFSSENQQFVSSTPFLGGQSC  66
E7F5T6      DANRE                  1 ---------------------------------------------------MALLGDQN-   8
A0A0G2KGP5  DANRE                 24 DARGFVLATP------MSHVFANNHWSFPNQP-FGLPCPAQSENQQS------CFNGVSD  70
A0A2R8PYX0  DANRE                  1 ------------------------------------------------------------   0
A8JUV0      SBNO DROME           236 --KGSNAPPPPPPSTAANSVRHSPTGGIPN--------P-----------GGNFFGGSAA 274
A8JUV0-3    SBNO DROME           188 ------------------------------------------------------------ 187

Q7TNB8      SBNO2 MOUSE           67 PETSYPTTATV---PSFFSKSSDFPQDPSCLEDLSNASV--FSSSVDSLSDIPDTPD--- 118
Q7TNB8-2    SBNO2 MOUSE           67 PETSYPTTATV---PSFFSKSSDFPQDPSCLEDLSNASV--FSSSVDSLSDIPDTPD--- 118
Q7TNB8-3    SBNO2 MOUSE            1 --------MSQ---LRFWLQFAALNKDPSCLEDLSNASV--FSSSVDSLSDIPDTPD---  44
Q9Y2G9      SBNO2 HUMAN ISO1      71 PDTSYAPVATA---SSLPPKTCDFAQDSSYFEDFSNISI--FSSSVDSLSDIVDTPD--- 122
Q9Y2G9-3    SBNO2 HUMAN ISO2      18 ----SAGTMSQ---LQLWLQFEALNKDSSYFEDFSNISI--FSSSVDSLSDIVDTPD---  65
A0A0G2JZZ8  SBNO2 RAT             71 PTPSFMNLDLSDIDFHELVRNGDFSQELPCTDEV--ASL--FSSPSDSLLEYPEP-S--- 122
D3ZDU8      SBNO2 RAT             67 PETSYPTTATV---PSFFSKSSDFPQDPSCLEDLSNASV--FSSSVDSLSDIPDTPD--- 118
E7F5T6      DANRE                  9 ---SFTVPCGI---------PTLGVQDPSCLEDLSNASV--FSSSVDSLSDIPDTPD---  51
A0A0G2KGP5  DANRE                 71 MHTDLLPQDLSNFDFSGL----DLPQDLSCLDDQ---LI--FSPQNDSLSEYAST-N--- 117
A0A2R8PYX0  DANRE                  1 --MSLMQIWTKLYAQLGR----PPPKDLSCLDDQ---LI--FSPQNDSLSEYAST-N---  45
A8JUV0      SBNO DROME           275 ASTSASAAN-FNMAASLL-------AQMSYAGGASQIRALKVAGNIGGVGNNQKPPPIAT 326
A8JUV0-3    SBNO DROME           188 ---------------------------VSYAGGASQIRALKVAGNIGGVGNNQKPPPIAT 220
                                                                              .:   ..: :  .

Q7TNB8      SBNO2 MOUSE          119 ---------FLQADSL-------NEVPTIWDV------STTSTTHDKLFIPSGPFSAP-- 154
Q7TNB8-2    SBNO2 MOUSE          119 ---------FLQADSL-------NEVPTIWDV------STTSTTHDKLFIPSGPFSAP-- 154
Q7TNB8-3    SBNO2 MOUSE           45 ---------FLQADSL-------NEVPTIWDV------STTSTTHDKLFIPSGPFSAP--  80
Q9Y2G9      SBNO2 HUMAN ISO1     123 ---------FLPADSL-------NQVSTIWDD------NPAPSTHDKLFQLSRPFAGF-- 158
Q9Y2G9-3    SBNO2 HUMAN ISO2      66 ---------FLPADSL-------NQVSTIWDD------NPAPSTHDKLFQLSRPFAGF-- 101
A0A0G2JZZ8  SBNO2 RAT            123 ---------GFGTDSLAQLTGEGPAAQMYWDI------PGHSQRPLN----LSAFPG--- 160
D3ZDU8      SBNO2 RAT            119 ---------FLPADSL-------NEVPTIWDV------STTSTTRDKLFLPSVPFSAL-- 154
E7F5T6      DANRE                 52 ---------FLPADSL-------NEVPTIWDV------STTSTTRDKLFLPSVPFSAL--  87
A0A0G2KGP5  DANRE                118 ---------FLNA-------DMAPTVPTIWDI------KTPAPVQES----SNRFQGLN- 150
A0A2R8PYX0  DANRE                 46 ---------FLNA-------DMAPTVPTIWDI------KTPAPVQES----SNRFQGLN-  78
A8JUV0      SBNO DROME           327 TPGSGGPAGGAPGSGVKGNNSMMEAVQKLIAMNPEYLTSGIPNTVFQMFMQSMQRPQATP 386
A8JUV0-3    SBNO DROME           221 TPGSGGPAGGAPGSGVKGNNSMMEAVQKLIAMNPEYLTSGIPNTVFQMFMQSMQRPQATP 280
                                                              .                    .

Q7TNB8      SBNO2 MOUSE          155 --EDPV--TSLSSTPLLISYQ-SH---SQPEEEEGEEEEETEELGHAETYADYVPSKSKI 206
Q7TNB8-2    SBNO2 MOUSE          155 --EDPV--TSLSSTPLLISYQ-SH---SQPEEEEGEEEEETEELGHAETYADYVPSKSKI 206
Q7TNB8-3    SBNO2 MOUSE           81 --EDPV--TSLSSTPLLISYQ-SH---SQPEEEEGEEEEETEELGHAETYADYVPSKSKI 132
Q9Y2G9      SBNO2 HUMAN ISO1     159 --EDFL--PS-HSTPLLVSYQ-EQSVQSQPEEEDEAEEEEAEELGHTETYADYVPSKSKI 212
Q9Y2G9-3    SBNO2 HUMAN ISO2     102 --EDFL--PS-HSTPLLVSYQ-EQSVQSQPEEEDEAEEEEAEELGHTETYADYVPSKSKI 155
A0A0G2JZZ8  SBNO2 RAT            161 -RLDEL--NAIL-SPSVAGFKAAAAPAPPPEEEEEADDEETEELGHADTYAEYKPSKSTI 216
D3ZDU8      SBNO2 RAT            155 --EDPV--TAVSSTPLLVSYQ-SH---SQPEEEEGEDEEEAEELGHAETYADYVPSKSKI 206
E7F5T6      DANRE                 88 --EDPV--TAVSSTPLLVSYQ-SH---SQPEEEEGEDEEEAEELGHAETYADYVPSKSKI 139
A0A0G2KGP5  DANRE                151 -SLDDI--SAIINTPPIGGY---QRPQTQPEEEEEVDVEEAEELGHVDTYADYKPSKSKI 204
A0A2R8PYX0  DANRE                 79 -SLDDI--SAIINTPPIGGY---QRPQTQPEEEEEVDVEEAEELGHVDTYADYKPSKSKI 132
A8JUV0      SBNO DROME           387 SPNQPMNPGAMVTSAAAA----AAHASAVAYVQQEEDEVDYEEMGVAETYADYWPAKLKL 442
A8JUV0-3    SBNO DROME           281 SPNQPMNPGAMVTSAAAA----AAHASAVAYVQQEEDEVDYEEMGVAETYADYWPAKLKL 336
                                        : :   :   :                  :: :   : **:* .:***:* *:* .:
```

Supplementary Figure 3: SBNO2 structural and phylogenetic analysis. (a) AF-Q9Y2G9-F1 alpha fold artificial intelligence-based prediction of the human SBNO2 ISO1 structure. The colour indicates the confidence of prediction. (b) Consurf-based prediction of SBNO2 phylogenetic conservation visualised on the AF-Q9Y2G9-F1 model. (c) Visualisation of SBNO2 NTP hydrolase domain (purple) and Helicase C domain (turquoise), and (d) visualisation of differences in SBNO2 ISO1 and SBNO2 ISO2 protein sequence using the AF-Q9Y2G9-F1 model. (e) Phylogenetic tree of SBNO2 isoforms across selected species. (f) Multiple sequence alignment of protein sequences of select SBNO2 isoforms according to (e). Amino acid 1 – 212 of human SBNO2 ISO1 (QY92G9) and corresponding residues of selected species are shown.

Supplementary Figure 4

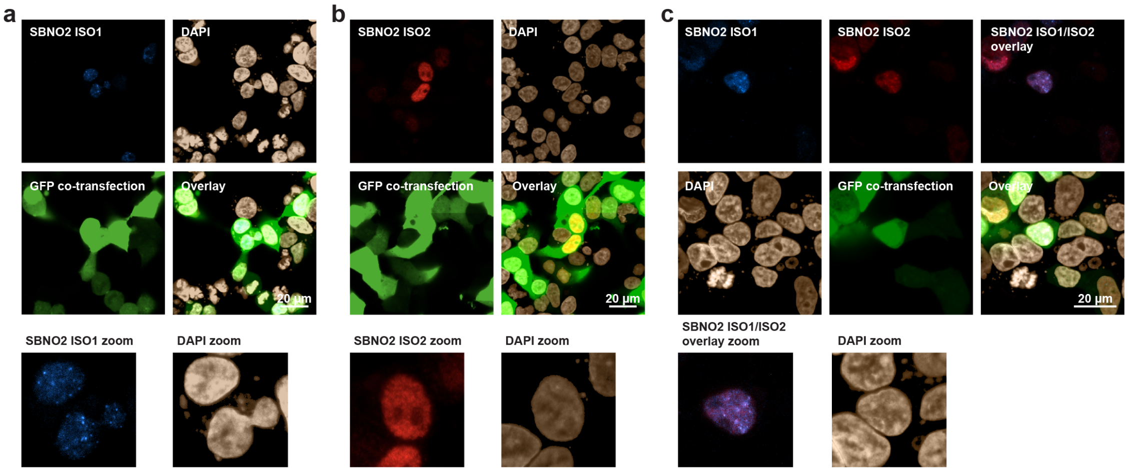

Supplementary Figure 4: Analyses of ectopic SBNO2 isoform expression and localization in HEK293 cells. (a-c) Confocal microscopy images of HEK293 cells expressing Myc-tagged SBNO2 ISO1 (cyan hot) (a), HA-tagged SBNO2 ISO2 (red) (b), and co-transfected HEK293 cells expressing both SBNO2 isoforms (c). Nuclear DAPI staining and co-transfected GFP fluorescence signals are shown in sepia and green colour respectively. Source data are provided as a Source Data file for Supplementary Figures 4a-c.

Supplementary Table 1: RISK cohort patient information.

	No IBD			CD non inflamed			CD microscopic inflammation			CD macroscopic inflammation		
	**Age**	**Sex**	**BMI**	**Age**	**Sex**	**BMI**	**Age**	**Sex**	**BMI**	**Age**	**Sex**	**BMI**
**Minimum**	4.5		12.59	7.833		13.61	2.25		12.98	5.083		12.78
**1st Quartile**	10.44		16.52	10.38		14.29	10.77		16.03	10.67		14.69
**Median**	12.88		19.54	11.5		16.3	13.04		17.8	12.42		16.43
**Mean**	12.37		20.49	12.07		16.86	12.67		18.45	12.45		17.36
**3rd Quartile**	14.85		23.51	13.79		18.4	15.21		20.35	14.58		19.01
**Maximum**	18		33.24	16.58		23.75	16.92		25.27	16.83		36.46
**Male**		n=38			n=17			n=14			n=93	
**Female**		n=28			n=8			n=10			n=50	

Supplementary Figure 5

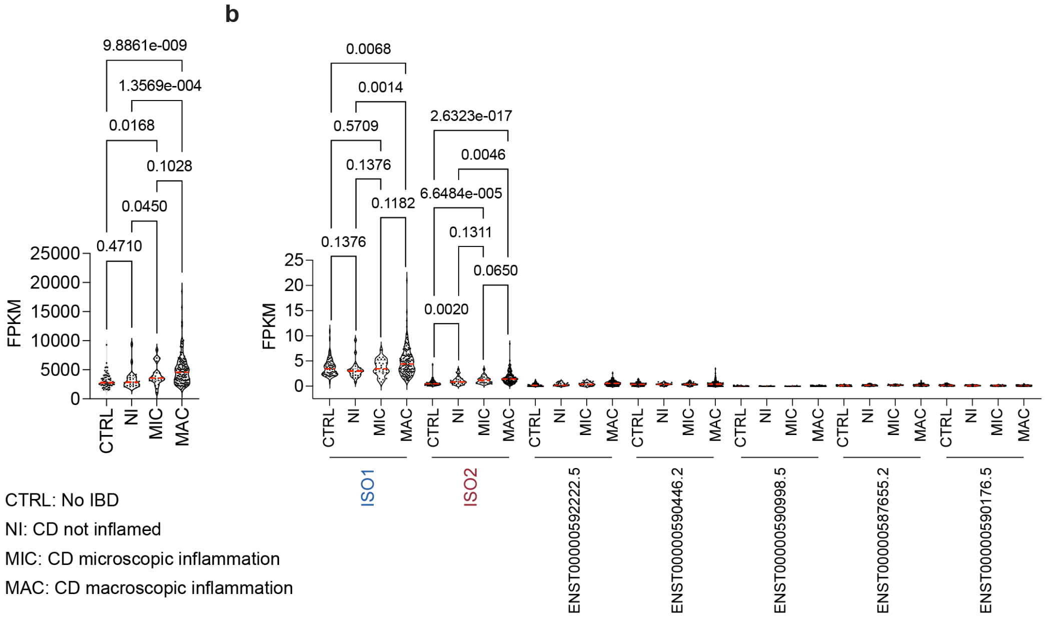

CTRL: No IBD

NI: CD not inflamed

MIC: CD microscopic inflammation

MAC: CD macroscopic inflammation

Supplementary Figure 5: *SBNO2* gene and isoform-level expression in intestinal biopsies from healthy individuals and patients with CD. (a) RNA sequencing-based estimation of *SBNO2* gene expression (non-parametric, two-sided, Kruskal-Wallis test) and (b) *SBNO2* isoforms expression in the RISK cohort data expressed as fragments per kilobase of exon per million mapped fragments (FPKM) (non-parametric, two-sided, Kruskal-Wallis test). Source data are provided as a Source Data file for Supplementary Figures 5a and b.

Supplementary Figure 6

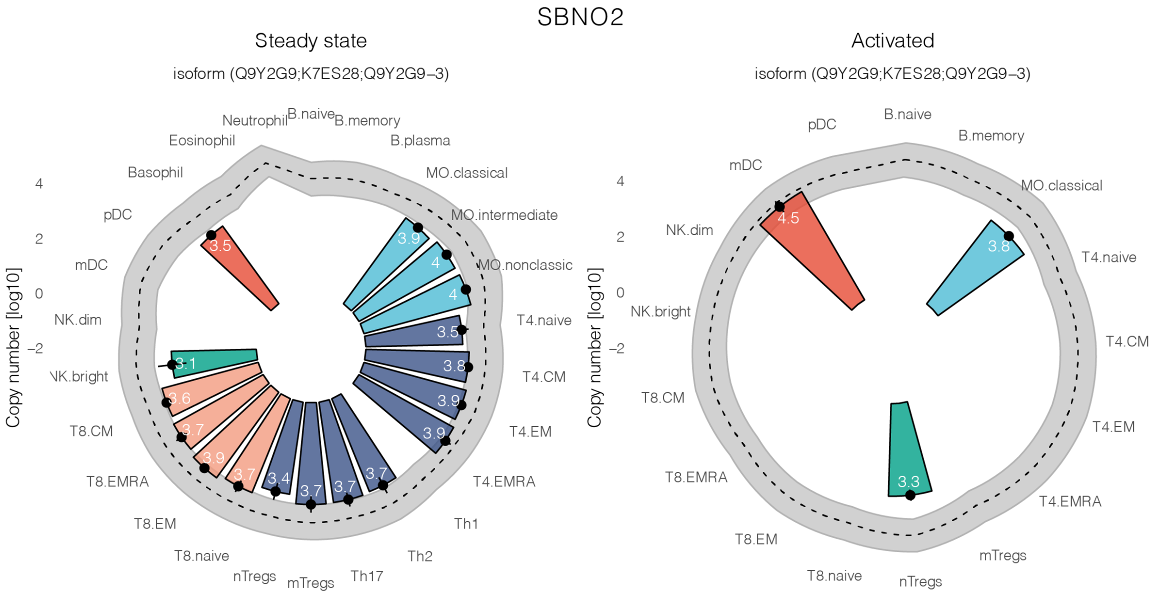

Supplementary Figure 6: Analyses of SBNO2 protein expression peripheral blood immune cells. Absolute protein copy numbers (log10) from high-sensitivity mass spectrometry quantification of SBNO2 in FACS-sorted peripheral blood immune cell types, in steady state and following stimulation (activated)[27]. The grey ribbon corresponds to the interquartile range of protein expression for all analytes in each cell.

Supplementary Figure 7

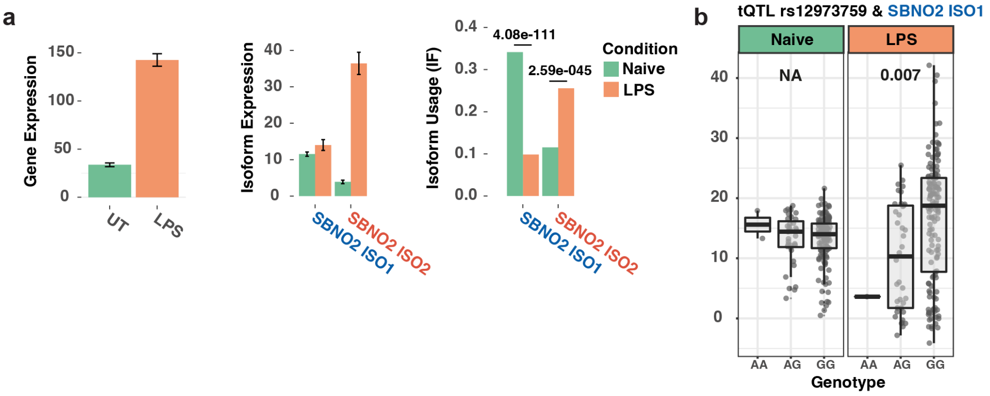

Supplementary Figure 7: *SBNO2* gene and isoform level expression in primary human monocytes. (a) *SBNO2* gene expression, *SBNO2* ISO1 and *SBNO2* ISO2 expression, and *SBNO2* ISO1 and *SBNO2* ISO2 isoform usage in resting and 24 hrs LPS-stimulated primary human monocytes (n = 176, we applied the IsoformSwitchAnalyzeR tool to analyse isoform usage in naïve and treated monocytes with LPS. Isoform usage refers to the fraction value of the mean isoform expression given the mean expression of the corresponding gene in a setting with k biological replicates). (b) Example of a variant (rs12973759) with a tQTL specifically affecting *SBNO2* ISO1 expression in LPS-stimulated $CD14^+$ monocytes. Box plots depict the interquartile range as the lower and upper bounds, respectively. The whiskers represent minimum and maximum, and the centre depicts the median. eQTL analysis was performed with the FastQTL and QTLtools using a linear regression. To allow comparison with output of the regression model the optimal number of PC was used to regress out expression changes attributable to the effect of the non-genetic covariates in local association plots. Source data are provided as a Source Data file for Supplementary Figures 7a and b.

Supplementary Table 2: Information on healthy individuals recruited via the Oxford Biobank (n = 176).

	Age	Sex	BMI
**Minimum**	31		17.43
**1st Quartile**	35		22.19
**Median**	49		24.29
**Mean**	47.22		25.19
**3rd Quartile**	57		26.98
**Maximum**	66		43.04
**Male**		n=60	
**Female**		n=116	

Supplementary Figure 8

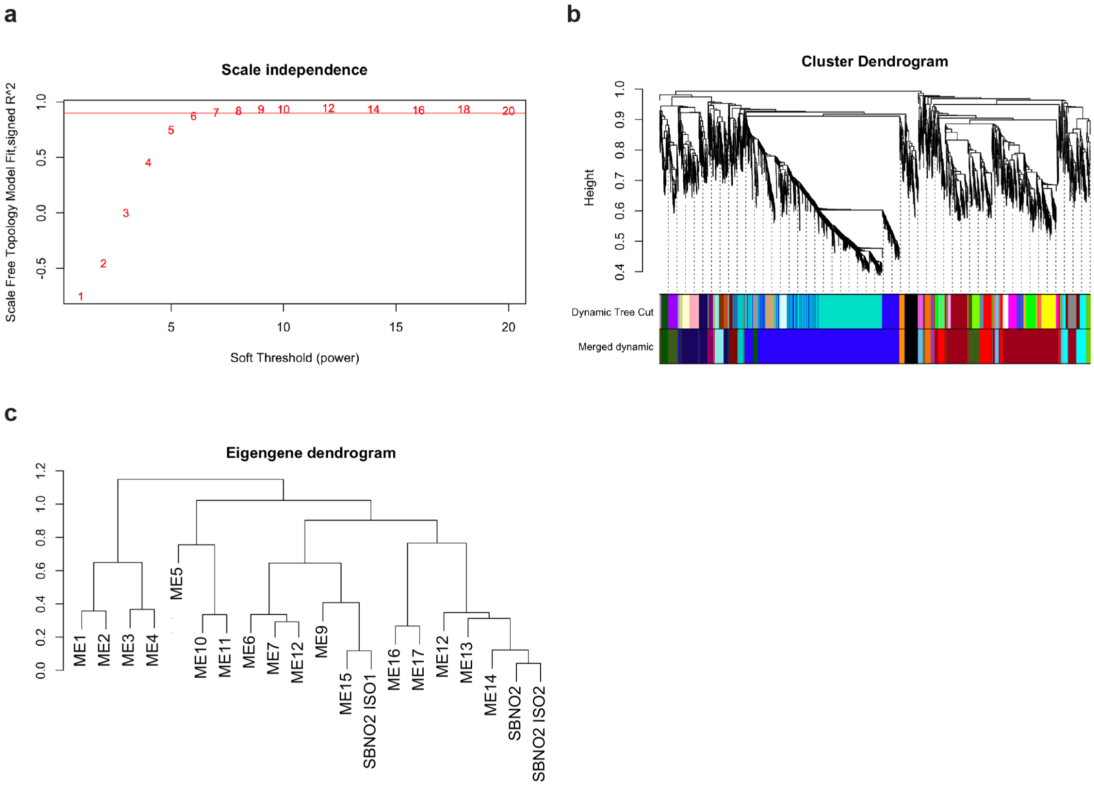

Supplementary Figure 8: WGCNA analysis of RNA-seq data generated from unstimulated, IL-10-, LPS, and LPS + aIL10R-stimulated MDM. (a) WGCNA soft power plot. (b) Cluster dendrogram. (c) Eigengene dendrogram including genes and transcripts of interest: *SBNO2*, *SBNO2* ISO1, and *SBNO2* ISO2.

Supplementary Table 3: Summary of MAGMA[33]-based gene-set heritability analysis.

			**IBD**				
VARIABLE	TYPE	NGENES	BETA	BETA_STD	SE	P	-LOG10(pvalue)
ME1	SET	918	0.091263	0.019998	0.031429	0.0018458	2.733815358
ME2	SET	235	0.05913	0.0066842	0.063221	0.17483	0.757384042
ME3	SET	252	0.080271	0.0093921	0.057515	0.081419	1.089274236
ME4	SET	305	-0.012925	-0.0016613	0.054697	0.5934	0.226652458
ME5	SET	53	0.23633	0.012752	0.13181	0.036496	1.437754732
ME6	SET	61	-0.0060427	-0.00034971	0.11719	0.52056	0.283529206
ME7	SET	118	-0.062614	-0.0050319	0.081982	0.77749	0.109305188
ME8	SET	50	-0.15116	-0.0079225	0.13264	0.87277	0.05910019
ME9	SET	120	0.21039	0.01705	0.089683	0.0094944	2.022532475
ME10	SET	153	0.13018	0.011902	0.077989	0.047542	1.322922552
ME11	SET	38	0.2006	0.0091689	0.14706	0.086274	1.064120066
ME12	SET	97	0.060824	0.0044344	0.094536	0.25999	0.585043356
ME13	SET	33	0.096503	0.0041109	0.18115	0.29712	0.527068114
ME14	SET	82	0.12018	0.0080594	0.11393	0.14575	0.836391437
ME15	SET	1438	0.1419	0.038324	0.026382	3.80E-08	7.419827998
ME16	SET	40	-0.041376	-0.0019401	0.14555	0.6119	0.213319547
ME17	SET	93	0.032885	0.0023478	0.095212	0.3649	0.437826137
			**CD**				
VARIABLE	TYPE	NGENES	BETA	BETA_STD	SE	P	-LOG10(pvalue)
ME1	SET	918	0.070448	0.015439	0.030936	0.011394	1.943323785
ME2	SET	235	0.057781	0.0065328	0.062689	0.17834	0.748751238
ME3	SET	252	0.076924	0.0090019	0.055966	0.084657	1.072337126
ME4	SET	305	-0.023835	-0.003064	0.054534	0.66896	0.17459985
ME5	SET	53	0.063144	0.0034076	0.1295	0.31292	0.504566678
ME6	SET	61	-0.15992	-0.0092565	0.11426	0.91917	0.036604159
ME7	SET	118	-0.0050225	-0.0004037	0.081024	0.52471	0.280080659
ME8	SET	50	-0.01468	-0.00076951	0.13234	0.54416	0.264273385
ME9	SET	120	0.12528	0.010154	0.090237	0.082528	1.08339868
ME10	SET	153	0.076462	0.0069914	0.075688	0.1562	0.80631897
ME11	SET	38	0.1205	0.0055087	0.14354	0.2006	0.697669071
ME12	SET	97	0.18849	0.013744	0.0879	0.016008	1.795662924
ME13	SET	33	0.38933	0.016588	0.18343	0.016907	1.771933447
ME14	SET	82	0.0042318	0.00028383	0.10963	0.4846	0.31461659
ME15	SET	1438	0.09979	0.026956	0.026036	6.36E-05	4.196816111
ME16	SET	40	0.049797	0.0023354	0.15539	0.37431	0.42676857
ME17	SET	93	0.16664	0.011899	0.092567	0.035927	1.444579046
			**UC**				
VARIABLE	TYPE	NGENES	BETA	BETA_STD	SE	P	-LOG10(pvalue)
ME1	SET	918	0.05496	0.012046	0.029513	0.031291	1.504580557
ME2	SET	235	0.0060121	0.00067979	0.058251	0.4589	0.338281942
ME3	SET	252	0.032884	0.0038485	0.052979	0.2674	0.572838597
ME4	SET	305	0.04416	0.0056773	0.052271	0.19911	0.700906928
ME5	SET	53	0.22172	0.011966	0.12522	0.038323	1.416540501
ME6	SET	61	0.039947	0.0023124	0.10657	0.35389	0.451131709
ME7	SET	118	0.031772	0.002554	0.078617	0.34306	0.464629917
ME8	SET	50	-0.15595	-0.0081757	0.12574	0.89256	0.04936258
ME9	SET	120	0.14046	0.011386	0.085852	0.050917	1.293137193
ME10	SET	153	0.10037	0.0091785	0.072839	0.084108	1.075162694
ME11	SET	38	0.14439	0.006601	0.1367	0.14544	0.837316134
ME12	SET	97	-0.11286	-0.0082303	0.087245	0.90209	0.044750131
ME13	SET	33	-0.038694	-0.0016487	0.17146	0.58927	0.229685668
ME14	SET	82	0.13138	0.0088125	0.10591	0.10741	0.968955283
ME15	SET	1438	0.078853	0.021302	0.024584	6.71E-04	3.17357531
ME16	SET	40	0.10553	0.0049498	0.1434	0.23089	0.636594876
ME17	SET	93	-0.00021387	-0.000015273	0.088658	0.50096	0.30019695

Supplementary Figure 9

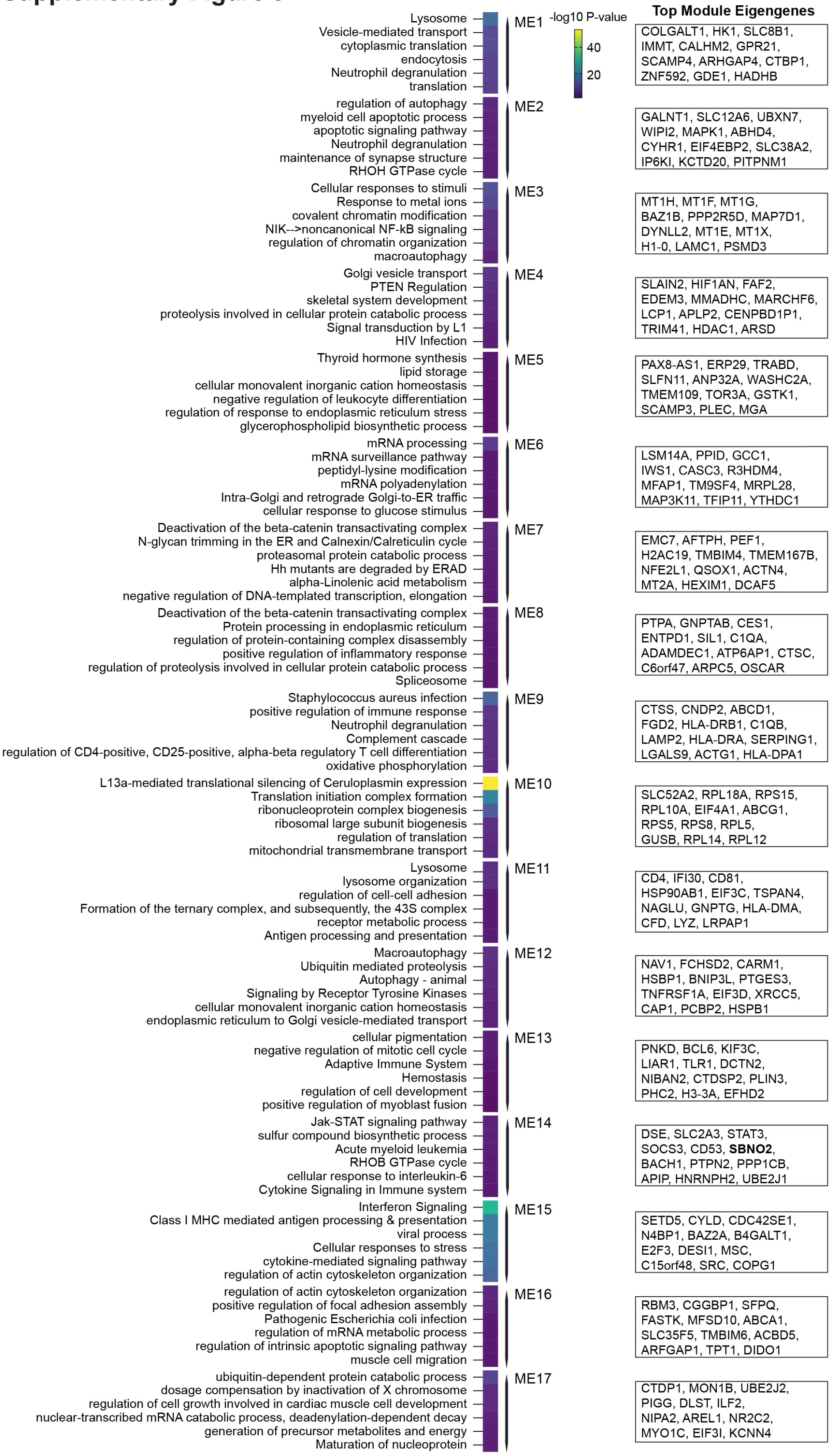

Supplementary Figure 9: Pathway analysis of WGCNA gene modules identified in unstimulated, IL-10-, LPS, and LPS + aIL10R-stimulated MDM. Metascape enrichment of KEGG, Reactome, and GO pathway terms based on those gene modules that were identified in unstimulated MDM and 8 hrs IL-10 (100 ng/mL), LPS (200 ng/mL), or LPS + aIL-10R (200 ng/mL, 10 µg/mL) stimulated MDM (hypergeometric test, Bonferroni correction, padj<0.05). Genes listed on the right represent the respective top 12 correlated module eigengenes. Source data are provided as a Source Data file for Supplementary Figure 9.

Supplementary Figure 10

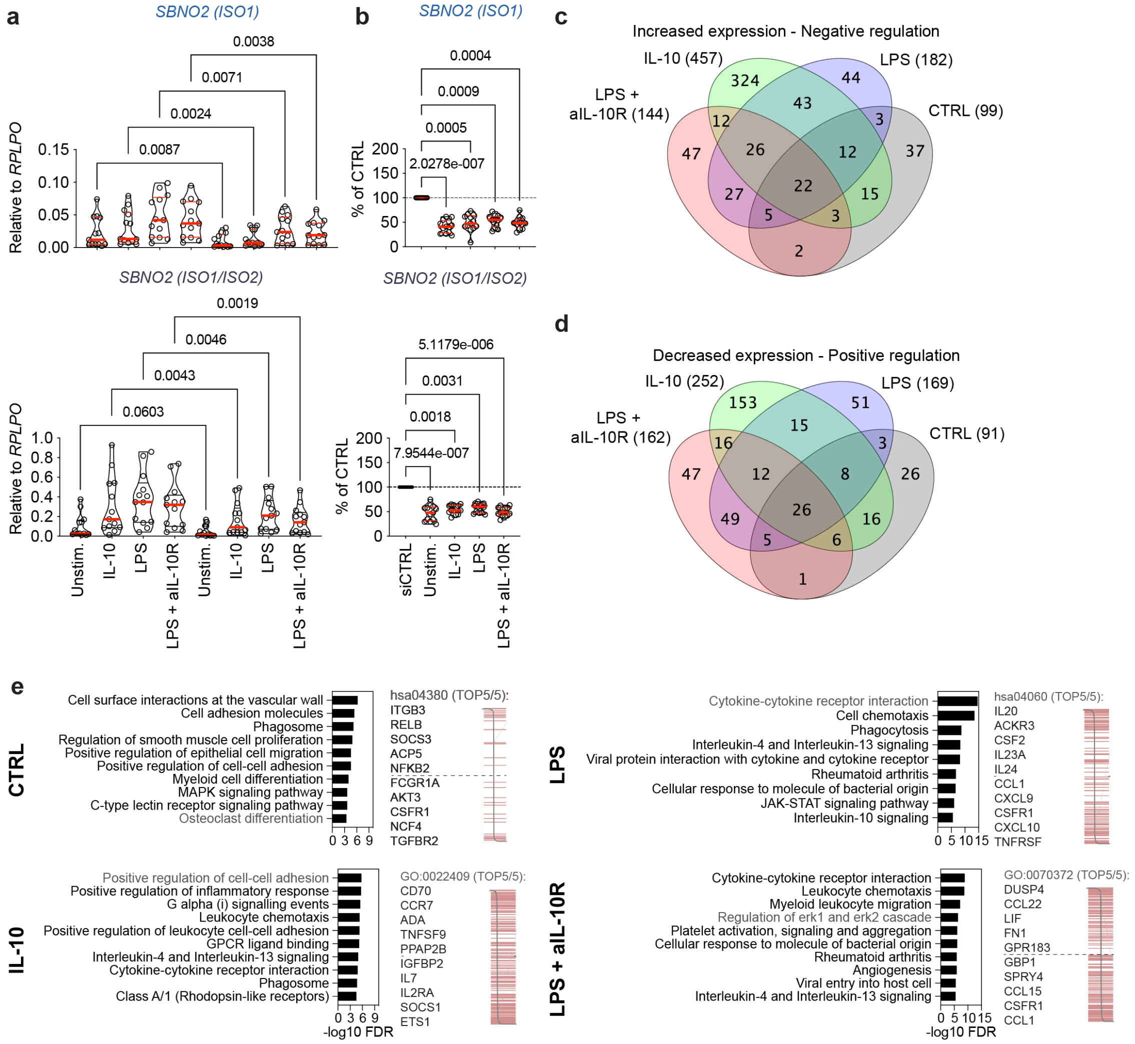

Supplementary Figure 10: siRNA-mediated knockdown of SBNO2 in primary human MDM. (a and b) RT-qPCR analysis of *SBNO2* ISO1 (a) and *SBNO2* ISO1/ISO2 (b) expression in primary human MDM shown as relative expression to *RPLPO* (left) and fold change to control siRNA-treated MDM (right) (Independent experiments/donors: n = 6/13; nonn-parametric, two-sided, Friedman test). (c and d) Venn diagrams show the numbers of significantly (log_2 fold change > 0.25, padj<0.05, FDR) differentially up-regulated (c) and down-regulated (d) genes upon knockdown of *SBNO2* in MDM (n=3) comparing unstimulated (CTRL), IL-10- (100 ng/mL), LPS- (200 ng/mL), or LPS+aIL-10R-treated (200 ng/mL, 10 µg/mL) conditions by RNA-seq and DESeq2 (n=3). (e) STRING functional enrichment analysis showing the top 10 enriched pathways (GO Biologic Process, KEGG Pathways, Reactome Pathways) with dual distribution based on DESeq2 differential expression ranking and functional enrichment analysis false discovery rate. Each one selected pathway is highlighted. The respective top 5 SBNO2 knockdown upregulated and top 5 SBNO2 knockdown downregulated (FDR) pathway genes are listed and the distribution according to log_2 fold change expression across the dataset visualized. Source data are provided as a Source Data file for Supplementary Figures 10a and b.

Supplementary Figure 11

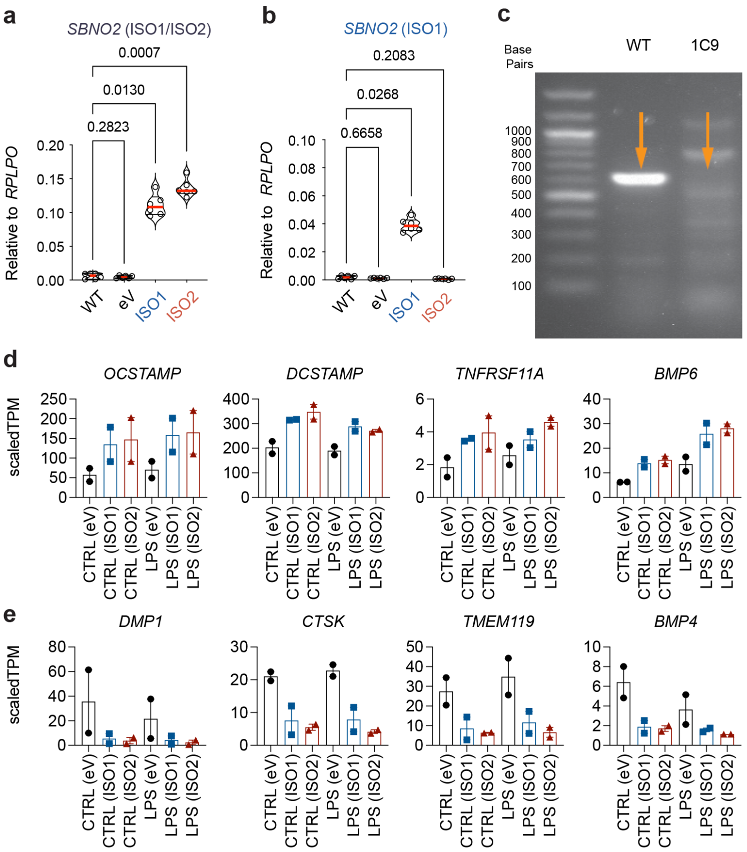

Supplementary Figure 11: Ectopic expression and CRISPR-Cas9-mediated knockout of SBNO2 in the THP-1 cell line. (a and b) RT-qPCR analysis of (a) *SBNO2* ISO1/ISO2, and (b) *SBNO2* ISO1 expression in lentivirus transduced, GFP^{+}-sorted, and PMA-differentiated THP-1 MDM (Independent experiments/replicates: n = 2/6, non-parametric, two-sided, Kruskal-Wallis test). (c) Agarose gel image showing the results from a PCR amplification of genomic DNA of the CRSPR-Cas9-targeted *SBNO2* region in WT THP-1 monocytes and a THP-1 single cell clone (1C9). (d) Expression of *OCSTAMP*, *DCSTAMP*, *TNFRSF11A*, and *BMP6* in lentivirus-transduced (empty vector (eV), SBNO2 ISO1 (ISO1), and SBNO2 ISO2 (ISO2)) THP-1 MDM in steady state or following 8 hrs stimulation with 200 ng/mL LPS measured by RNA-seq (n = 2). (e) Expression of *DMP1*, *CTSK*, *TMEM119*, and *BMP4* according to (d) (n = 2). Source data are provided as a Source Data file for Supplementary Figures 11a-e.
