# Supplementary material for: An isoform quantitative trait locus in SBNO2 links genetic susceptibility to Crohn’s disease with defective antimicrobial activity
Source: Nat Commun. 2024 May 28;15:4529. doi: 10.1038/s41467-024-47218-3 (PMC11133462; doi:10.1038/s41467-024-47218-3)
Supplement: Supplementary file 3 — Description of Additional Supplementary Files [file 41467_2024_47218_MOESM3_ESM.pdf]

Description of Additional Supplementary Files

File Name: Supplementary Data 1
Description: Monocyte-derived macrophages in vitro stimulation - RNA sequencing-based WGCNA modules.

File Name: Supplementary Data 2
Description: siRNA-mediated knockdown of SBNO2 in Monocyte-derived macrophages - RNA sequencing and DESeq2-based differentially expressed genes.

File Name: Supplementary Data 3
Description: siRNA-mediated knockdown of SBNO2 in Monocyte-derived macrophages - Functional pathway enrichment analysis of RNA sequencing and DESeq2-based differentially expressed genes - Unstimulated condition.

File Name: Supplementary Data 4
Description: siRNA-mediated knockdown of SBNO2 in Monocyte-derived macrophages - Functional pathway enrichment analysis of RNA sequencing and DESeq2-based differentially expressed genes - IL-10-stimulated condition.

File Name: Supplementary Data 5
Description: siRNA-mediated knockdown of SBNO2 in Monocyte-derived macrophages - Functional pathway enrichment analysis of RNA sequencing and DESeq2-based differentially expressed genes - LPS-stimulated condition.

File Name: Supplementary Data 6
Description: siRNA-mediated knockdown of SBNO2 in Monocyte-derived macrophages - Functional pathway enrichment analysis of RNA sequencing and DESeq2-based differentially expressed genes - LPS+aIL-10R-stimulated condition.
