# Supplementary material for: An isoform quantitative trait locus in SBNO2 links genetic susceptibility to Crohn’s disease with defective antimicrobial activity
Source: Nat Commun. 2024 May 28;15:4529. doi: 10.1038/s41467-024-47218-3 (PMC11133462; doi:10.1038/s41467-024-47218-3)
Supplement: Supplementary file 10 — Reporting Summary [file 41467_2024_47218_MOESM10_ESM.pdf]

nature portfolio

Corresponding author(s): Dominik Aschenbrenner
Holm H. Uhlig

Last updated by author(s): Feb 3, 2024

Reporting Summary

Nature Portfolio wishes to improve the reproducibility of the work that we publish. This form provides structure for consistency and transparency in reporting. For further information on Nature Portfolio policies, see our Editorial Policies and the Editorial Policy Checklist.

Statistics

For all statistical analyses, confirm that the following items are present in the figure legend, table legend, main text, or Methods section.

n/a	Confirmed	
☐	☒	The exact sample size (*n*) for each experimental group/condition, given as a discrete number and unit of measurement
☐	☒	A statement on whether measurements were taken from distinct samples or whether the same sample was measured repeatedly
☐	☒	The statistical test(s) used AND whether they are one- or two-sided<br>*Only common tests should be described solely by name; describe more complex techniques in the Methods section.*
☐	☒	A description of all covariates tested
☐	☒	A description of any assumptions or corrections, such as tests of normality and adjustment for multiple comparisons
☐	☒	A full description of the statistical parameters including central tendency (e.g. means) or other basic estimates (e.g. regression coefficient) AND variation (e.g. standard deviation) or associated estimates of uncertainty (e.g. confidence intervals)
☐	☒	For null hypothesis testing, the test statistic (e.g. *F*, *t*, *r*) with confidence intervals, effect sizes, degrees of freedom and *P* value noted<br>*Give P values as exact values whenever suitable.*
☒	☐	For Bayesian analysis, information on the choice of priors and Markov chain Monte Carlo settings
☒	☐	For hierarchical and complex designs, identification of the appropriate level for tests and full reporting of outcomes
☐	☒	Estimates of effect sizes (e.g. Cohen's *d*, Pearson's *r*), indicating how they were calculated

Our web collection on statistics for biologists contains articles on many of the points above.

Software and code

Policy information about availability of computer code

Data collection	Preparative flow cytometry:<br>- BD FACSDiva v9.0<br><br>Microscopy:<br>- ZEN2011 software<br><br>qPCR:<br>- BIO-RAD FX96 Touch Real-Time PCR Detection System - CFX Maestro Software
Data analysis	Analytical and preparative flow cytometry:<br>- Becton Dickinson FlowJo 10.8.1<br><br>Microscopy:<br>- ImageJ2, Version 2.3.0/153f<br><br>Graphing and statistical analyses:<br>- GraphPad Prism, Version 9 and 10<br>- Microsoft Excel for Mac, Version 16.16.27<br>- RStudio, Version 3.6.3<br>- R, Version 1.1.456

RNA-sequencing analysis:
- Salmon R package, Version 1.2.1
- tximport R package, Version 1.30.0
- AnnotationDbi R package, Version 1.48
- DESeq2 R package, Version 1.42.0
- apeglm R package, Version 1.24.0
- ggplot2 R package, Version 3.3.1
- pheatmap R package, Version 1.0.12
- STRING - Functional Enrichment Analysis: https://string-db.org/

Analysis of differential transcript expression and transcript usage in monocyte-derived macrophages:
- Salmon R package, Version 1.2.1
- tximport R package, Version 1.30.0
- Genomic Feattures R package, Version 1.38.0
- DRIMseq R package, Version 1.14.0
- DEXseq R package, Version 1.32.0
- stageR R package, Version 1.8.0

WGCNA analysis:
- WGCNA R package, Version 1.69
- Metascape platform: https://metascape.org

MAGMA analysis:
- MAGMA, Version 1.10
- Annovar package, avsnp147, hg38, https://annovar.openbioinformatics.org

Monocyte eQTL, isoQTL and tQTL analysis:
- stats R package, Version 4.3.2
- IsoformSwitchAnalyzeR, Version 2.3.0
- FastQTL, Version 2.0
- QTLtools, Version 1.2
- ggpubr, Version 0.2
- ggplot2 R package, Version 3.3.1

Online resources:
- gnomAD: https://gnomad.broadinstitute.org/
- GTEx: https://gtexportal.org/home/
- GWAS Catalog: https://www.ebi.ac.uk/gwas/
- IBD Exomes Browser: https://dmz-ibd.broadinstitute.org/
- LDlink: https://ldlink.nci.nih.gov
- Open Targets Genetics: https://genetics.opentargets.org/
- PhenoScanner: http://www.phenoscanner.medschl.cam.ac.uk/
- SNPnexus: https://www.snp-nexus.org/v4/

For manuscripts utilizing custom algorithms or software that are central to the research but not yet described in published literature, software must be made available to editors and reviewers. We strongly encourage code deposition in a community repository (e.g. GitHub). See the Nature Portfolio guidelines for submitting code & software for further information.

Data

Policy information about availability of data

All manuscripts must include a data availability statement. This statement should provide the following information, where applicable:

- Accession codes, unique identifiers, or web links for publicly available datasets
- A description of any restrictions on data availability
- For clinical datasets or third party data, please ensure that the statement adheres to our policy

All sequencing data will be made freely available to organizations and researchers to conduct research following the UK Policy Framework for Health and Social Care Research via a data access agreement. Sequence data related to eQTL studies have been deposited at the European Genome–Phenome Archive, which is hosted by the European Bioinformatics Institute and the Centre for Genomic Regulation under accession no. EGAN00002778798. Raw RNA sequencing data (siRNA-mediated knockdown of SBNO2 in CD14+ MDM, and ectopic expression of SBNO2 isoforms in THP-1 MDM) from this study have been deposited at the European Genome–Phenome Archive, which is hosted by the European Bioinformatics Institute and the Centre for Genomic Regulation under the dataset ID EGAD50000000264 . RNA sequencing data for the RISK study is deposited in the SRA database SUB6656230. Source data are provided with this paper.

Research involving human participants, their data, or biological material

Policy information about studies with human participants or human data. See also policy information about sex, gender (identity/presentation), and sexual orientation and race, ethnicity and racism.

Reporting on sex and gender	Sex was determined based on self-reporting. Number of male and female donors for the RISK cohort analysis and healthy donors from the Oxford biobank are reported in Extended Data Tables 1 and 2 respectively. No sex-based analyses have been

performed since these are out of scope for this study.

Reporting on race, ethnicity, or other socially relevant groupings	NA
Population characteristics	The covariate relevant characteristics that have been recorded, Age, Sex, and BMI of the RISK cohort and healthy volunteers from the Oxford biobank are summarized in Extended Data Tables 1 and 2 respectively.<br>RISK cohort detailed information is available at: https://clinicaltrials.gov/study/NCT00790543.<br>Healthy volunteers of European ancestry samples were collected via the Oxford biobank (www.oxfordbiobank.org.uk) with full ethical approval and written informed consent.
Recruitment	Individuals previously enrolled in the RISK cohort study or the Oxford Biobank prior to conception of this study. Healthy donors were recruited via the Oxford gastrointestinal biobank (11/YH/0020 and 16/YH/0247). All healthy volunteers provided written informed consent. There were no self-selection biases other than being likely to donate to a biobank and living in the vicinity of Oxford and we have no evidence to think that these, if present, would influence results.
Ethics oversight	Experiments were carried out with Research Ethics Board (REB) approval from the Oxford IBD cohort study (Rare disease subproject and Research Ethics Committee Reference: 09/H1204/30), and with approval from the Oxford Research Ethics Committee (REC): Analysis of the relationship between genetic diversity and gene expression in human peripheral blood mononuclear cells taken from healthy volunteers (06/Q1605/55).

Note that full information on the approval of the study protocol must also be provided in the manuscript.

Field-specific reporting

Please select the one below that is the best fit for your research. If you are not sure, read the appropriate sections before making your selection.

☒ Life sciences ☐ Behavioural & social sciences ☐ Ecological, evolutionary & environmental sciences

For a reference copy of the document with all sections, see nature.com/documents/nr-reporting-summary-flat.pdf

Life sciences study design

All studies must disclose on these points even when the disclosure is negative.

Sample size	The sample size is sufficient for eQTL analysis in keeping with published literature, it was not estimated. The sample size provided power to detect eQTL effects with MAF of 0.05 with ~80% power and is consistent with those previously published.<br><br>1. Kim, S. et al. Characterizing the genetic basis of innate immune response in TLR4-activated human monocytes. Nat Commun 5, 5236 (2014).<br>2. Gilchrist, J. J. et al. Natural Killer cells demonstrate distinct eQTL and transcriptome-wide disease associations, highlighting their role in autoimmunity. Nat. Commun. 13, 4073 (2022).
Data exclusions	No data were excluded from analysis.
Replication	Biological and technical replicates are described in figure legends. The eQTL cohort is not replicated but results are iin allignment with previously published eQTL.
Randomization	The RISK study is an observational prospective cohort study with the aim to identify risk factors that predict complicated course in pediatric patients with Crohn's disease. The RISK study recruited treatment-naive patients with a suspected diagnosis of Crohn's disease. The Paris modification of the Montreal classification were used to classify patients according to disease behaviour (non-complicated B1 disease (non-stricturing, non-penetrating disease); complicated disease, composed of B2 (stricturing) and/or B3 (penetrating) behaviour) as well as disease location (L1, ileal only, L2, colonic only, L3, ileocolonic and L4, upper gastrointestinal tract). Individuals without ileal inflammation were classified as non-IBD controls. Patients with Crohn's disease were followed over a period of 3 years. Patients were largely of European (85.7%) and African (4.1%) ancestry. All covariates used are listed in the methods. For eQTL studies samples were not allocated into experimental groups. All participants gave samples for all experimental groups, all allocation is random and covariates are controlled through the use of principal components in the eQTL analysis.
Blinding	eQTL analysis was performed blinded as to the genotype and any other covariate.

Reporting for specific materials, systems and methods

We require information from authors about some types of materials, experimental systems and methods used in many studies. Here, indicate whether each material, system or method listed is relevant to your study. If you are not sure if a list item applies to your research, read the appropriate section before selecting a response.

Materials & experimental systems

n/a	Involved in the study	
☐	☒	Antibodies
☐	☒	Eukaryotic cell lines
☒	☐	Palaeontology and archaeology
☒	☐	Animals and other organisms
☒	☐	Clinical data
☒	☐	Dual use research of concern
☒	☐	Plants

Methods

n/a	Involved in the study	
☒	☐	ChIP-seq
☒	☐	Flow cytometry
☒	☐	MRI-based neuroimaging

Antibodies

Antibodies used

Immunofluorescence:
- anti-c-Myc (Biolegend; Cat.# 626810; clone: 9E10, dilution: 1:100, https://www.biolegend.com/en-gb/products/alexa-fluor-647-anti-c-myc-antibody-20210)
- anti-HA (Cell Signalling, Cat.# 55420; clone: C29F4, dilution: 1:100, https://www.cellsignal.com/products/antibody-conjugates/ha-tag-c29f4-rabbit-mab-alexa-fluor-555-conjugate/55420)

Blocking:
- anti-IL-10R (Biolegend; Cat.# 308807; clone: 3F9, 10 µg/mL, https://www.biolegend.com/de-at/products/ultra-leaf-purified-anti-human-cd210-il-10-r-antibody-17175)
https://www.biolegend.com/de-de/products/leaf-low-endotoxin--azide-freepurified-anti-mouse-cd210-il-10-r-antibody-1512

Validation

All antibodies used in this study have been validated by the manufacturer. Immunofluorescence antibodies were additionally validated on target expressing and non-expressing cells.

Eukaryotic cell lines

Policy information about cell lines and Sex and Gender in Research

Cell line source(s)

HEK293 (ATCC; Cat.# CRL-1573, https://www.atcc.org/)
THP1 (ATCC; Cat.# TIB-202, https://www.atcc.org/)

Authentication

Authentication was performed by the commercial provider and by visual inspection following defrosting and expansion (https://www.atcc.org/services/cell-authentication)

Mycoplasma contamination

Mycoplasma tests were performed after defrosting and expansion and found negative. In addition, cell cultures were regularly tested for the presence of Mycoplasma and found negative.

Commonly misidentified lines
(See ICLAC register)

None

Plants

Seed stocks

NA

Novel plant genotypes

NA

Authentication

NA
